# “iPSC-derived liver organoids and inherited bleeding disorders: Potential and future perspectives”

**DOI:** 10.3389/fphys.2023.1094249

**Published:** 2023-01-13

**Authors:** Giacomo Roman, Benedicte Stavik, Knut H. Lauritzen, Per Morten Sandset, Sean P. Harrison, Gareth J. Sullivan, Maria Eugenia Chollet

**Affiliations:** ^1^ Department of Hematology, Oslo University Hospital, Oslo, Norway; ^2^ Research Institute of Internal Medicine, Oslo University Hospital, Oslo, Norway; ^3^ Institute of Clinical Medicine, University of Oslo, Oslo, Norway; ^4^ Department of Pediatric Research, Oslo University Hospital, Oslo, Norway; ^5^ Department of Immunology, Institute of Clinical Medicine, University of Oslo, Oslo, Norway

**Keywords:** bleeding disorders, coagulation factor deficiencies, induced pluripotent stem cells, liver organoids, genome editing, CRISPR, cell therapy, disease modeling

## Abstract

The bleeding phenotype of hereditary coagulation disorders is caused by the low or undetectable activity of the proteins involved in hemostasis, due to a broad spectrum of genetic alterations. Most of the affected coagulation factors are produced in the liver. Therefore, two-dimensional (2D) cultures of primary human hepatocytes and recombinant overexpression of the factors in non-human cell lines have been primarily used to mimic disease pathogenesis and as a model for innovative therapeutic strategies. However, neither human nor animal cells fully represent the hepatocellular biology and do not harbor the exact genetic background of the patient. As a result, the inability of the current *in vitro* models in recapitulating the *in vivo* situation has limited the studies of these inherited coagulation disorders. Induced Pluripotent Stem Cell (iPSC) technology offers a possible solution to overcome these limitations by reprogramming patient somatic cells into an embryonic-like pluripotent state, thus giving the possibility of generating an unlimited number of liver cells needed for modeling or therapeutic purposes. By combining this potential and the recent advances in the Clustered Regularly Interspaced Short Palindromic Repeats (CRISPR)/Cas9 technology, it allows for the generation of autologous and gene corrected liver cells in the form of three-dimensional (3D) liver organoids. The organoids recapitulate cellular composition and organization of the liver, providing a more physiological model to study the biology of coagulation proteins and modeling hereditary coagulation disorders. This advanced methodology can pave the way for the development of cell-based therapeutic approaches to treat inherited coagulation disorders. In this review we will explore the use of liver organoids as a state-of-the-art methodology for modeling coagulation factors disorders and the possibilities of using organoid technology to treat the disease.

## 1 Introduction

**FIGURE 1:**
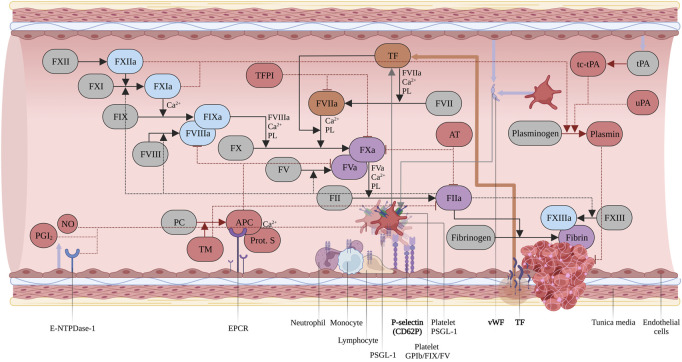
Coagulation cascade. Intrinsic pathway in light blue, extrinsic pathway in orange, common pathway in purple. Inhibitors and modulators of hemostasis in red. Zymogens in grey. Abbreviations: Tissue Factor Protein Inhibitor (TFPI); Phospholipids (PL); Two-chains, tissue-type Plasminogen Activator (tc-tPA); Urokinase Plasminogen Activator (uPA); Protein C (PC); activated Protein C (APC); Thrombomodulin (TM); Prostaglandin I_2_ (PGI_2_); Nitric Oxide (NO). Created with BioRender.com.

The hemostatic system is a conserved machinery encompassing four different compartments: the coagulation system, the vascular endothelium, platelets, and the fibrinolytic system, interacting in a tightly coordinated manner to achieve clot formation and restoration of vascular integrity. Upon vessel injury, the trauma results in endothelial cell and platelet activation *via* P-selectin translocation, respectively from the Weibel-Palade Bodies and the α-granule storages. This interaction promotes leukocyte rolling on activated endothelial cell, thus improving the localization and formation of the thrombus. In addition, the platelet adhesion at the site of injury is sustained by the von Willebrand Factor (vWF), which binds the platelet membrane glycoprotein (GP) Ibα and the exposed sub-endothelial connective tissue (Bhagavan and Ha, 2015). Then, the formation of platelet-leukocyte aggregates provides the proper environment for Tissue Factor (TF), exposed by the damaged vascular smooth muscle cells, pericytes and adventitial fibroblasts, to activate Factor VII (FVII) and initiate the coagulation processes. The pathways responsible for coagulation are the intrinsic and the extrinsic pathways, comprising multiple enzymatic reactions in which coagulation factors, cell membrane phospholipids, vitamin K and Ca^2+^ participate (Figure 1). Despite different initiation mechanisms, both pathways converge on a final common cascade, leading to thrombin activation, fibrin clot formation/stabilization, and inhibition of fibrinolysis (Sturgess, 2014).

## 2 The blood coagulation system

Upon vessel injury FVII binds tissue factor (TF) exposed in the sub-endothelium. The TF/activated FVII (FVIIa) complex generates the small amount of thrombin necessary to promote platelet, Factor XI (FXI), Factor V (FV) and Factor VIII (FVIII) further activation. For this reason, it is considered to be the key physiological activator of *in vivo* initiation of coagulation (Hoffman, 2003; MacKman, 2009; Smith, 2009). Upon dissociation from vWF and activation by thrombin, FVIIIa associates with FIXa to form the tenase complex, on a membrane surface which is provided by platelets, microparticles, and endothelial cells which leads to a large-scale generation of FXa (Baugh et al., 1998). Then, FXa, FVa and Ca^2+^ ions associate to form the Prothrombinase complex, producing the “Thrombin burst” necessary for a rapid conversion of Fibrinogen to Fibrin and activating FXIII, which results in the definitive clot stabilization (Orlova et al., 2013). The coagulation system is meticulously balanced to prevent pathophysiological clotting (thrombosis) or insufficient response (bleeding).

## 3 Inherited bleeding disorders

Inherited bleeding disorders represent a highly heterogeneous and complex group of conditions centered on impaired coagulation competence. They are caused by genetic mutations in the genes encoding the pro-coagulant factors, and deficiencies in almost all factors have been observed (Table 1). Based on the residual antigen and activity levels of the factor, they are clinically classified into a mild, moderate, and severe hemorrhagic phenotype. As a result of the tightly regulated and balanced nature of hemostasis, the clinical phenotype of severe, coagulation factor deficiencies could be significantly improved even by a small increase in terms of protein activity. This present review will focus on the most prevalent liver-synthesized coagulation factor deficiencies, Hemophilia A and B, and FVII deficiency.

**TABLE 1:** Overview of the coagulation factors and the major associated disorders. Gene sizes and chromosomal locations from UCSC Genome Browser (http://genome.ucsc.edu/).

Name	Mass (kDa)	Plasma conc. (mg/dL)	Function	Disorder	Gene location and inheritance	Incidence	Gene size (Kb)	OMIM #	Plasma half-life (h)	References
Fibrinogen (Factor I)	340	200–400	Bridging ligand for platelet GP IIb/IIIa, thrombin substrate in the establishment of fibrin clot	Afibrinogenemia	4q31.3 - 4q32.1; Autosomal recessive or dominant	1:1,000,000	FGA (7.6) FGB (8.1) FGG (8.5)	202400	72–120	Marín-García, (2007); Bolliger et al. (2010); Norström and Escolar, (2011); Palta et al. (2014); Winter et al. (2020)
Prothrombin (Factor II)	72	10–15	Conversion of fibrinogen into fibrin, activation of FV, FVIII and FXIII	FII deficiency	11p11.2; Autosomal recessive	1:2,000,000	F2 (20.3)	17930	60–70	Marín-García, (2007); Bolliger et al. (2010); Norström and Escolar, (2011); Palta et al. (2014); Winter et al. (2020)
Tissue factor (Factor III)	47	-	Primary initiator of coagulation, complexing with FVIIa (TF/FVIIa) to activate FIX	-	1p21.3	-	F3 (12.4)	-	-	Marín-García, (2007); Bolliger et al. (2010); Norström and Escolar, (2011); Palta et al. (2014); Winter et al. (2020)
Labile factor (Factor V)	330	0,5–1	Cofactor for FXa in the prothrombinase complex	Parahemophilia	1q24.2; Autosomal recessive	1:1,000,000	F5 (72.3)	227400	12–36	Tracy et al. (1982); Marín-García, (2007); Bolliger et al. (2010); Norström and Escolar, (2011); Winter et al. (2020)
Proconvertin (Factor VII)	50	0,05	Complex with FIII and Ca^2+^ to form FXa	FVII deficiency	13q34; Autosomal recessive	1:500,000	F7 (14.2)	227500	3–6	Rao, (2006); Marín-García, (2007); Bolliger et al. (2010); Norström and Escolar, (2011); Winter et al. (2020)
Antihemophilic factor (Factor VIII)	285	0,01–0,02	Cofactor of the tenase complex generating FXa	Hemophilia A	Xq28; X-linked recessive	1:10,000	F8 (186)	306700	8–12	Marín-García, (2007); Bolliger et al. (2010); Norström and Escolar, (2011); Winter et al. (2020)
Christmas factor (Factor IX)	57	0,3–0,5	Complexing with FVIII to form the tenase complex and activate FX	Hemophilia B	Xq27.1-27.2; X-linked recessive	1:50,000–60,000	F9 (33)	306900	18–24	Marín-García, (2007); Bolliger et al. (2010); Norström and Escolar, (2011); Winter et al. (2020)
Stuart–Prower factor (Factor X)	59	0,8–1	Prothrombin activation, in presence of FVa, Ca^2+^ and phospholipids	FX deficiency	13q34;Autosomal recessive	1:500,000–1,000,000	F10 (22)	227600	30–40	Marín-García, (2007); Bolliger et al. (2010); Norström and Escolar, (2011); Winter et al. (2020)
Plasma thromboplastin antecedent (Factor XI)	160	0,48–0,7	Activation of FIX	FXI deficiency	4q35.2; Autosomal recessive or dominant	1:1,000,000	F11 (22,6)	264900	52–80	Marín-García, (2007); Bolliger et al. (2010); Norström and Escolar, (2011); Winter et al. (2020)
Hageman factor (Factor XII)	80	3–4	Activator of FXI, FVII and prekallikrein	-	5q33	-	F12 (12)	-	60	Marín-García, (2007); Bolliger et al. (2010); Norström and Escolar, (2011); Winter et al. (2020)
Laki–Lorand, fibrin stabilizing factor (Factor XIII)	320	1–3	Fibrin clot stabilization via cross-linking transamidation	FXIII deficiency	6p25.1 (F13A1); 1q31 – 32.1 (F13B); Autosomal recessive	1:2,000,000–5,000,000	F13A1 (160); F13B (28)	613225; 613235	120–200	Marín-García, (2007); Bolliger et al. (2010); Norström and Escolar, (2011); Kattula et al. (2018)
von Willebrand factor (vWF)	500–20′000	0,1	Mediating platelet adhesion and functioning as a carrier for FVIII	Von Willebrand disease	12p13.31; Autosomal recessive or dominant	1:5000–10,000	vWF (178)	193400; 613554; 277480	10–24	Van Mourik and De Wit, (2001); Bolliger et al. (2010); Bhagavan and Ha, (2015)

### 3.1 Hemophilia A and B

Hemophilia A (OMIM #306700) is a monogenic, X-linked recessive bleeding disorder induced by the deficiency or reduced activity of FVIII, with a prevalence of 1:5000 amongst the male population (Graw et al., 2005). The most frequent genetic change that affects the *F8* gene, located at Xq28, is the intron 22 Inversion (Inv22), accounting for approximately 45% of patients with severe Hemophilia A (Oldenburg and El-Maarri, 2006). In cases of mild impairment, the residual FVIII activity ranges from 5% to 40%, and patients experience intense bleeding upon injuries, traumas, and surgeries. The hemorrhagic episodes of moderate and severe cases, correlating to 1%–5% and <1% FVIII activity, respectively, include spontaneous and recurrent bleedings as a result of minor endothelial injuries (White et al., 2001). The current Hemophilia A therapeutic treatment mainly consists of protein replacement, requiring frequent prophylactic or on-demand parenteral administrations of recombinant FVIII (r-FVIII) with an extended half-life or plasma-derived FVIII (Powell et al., 2012). However, neutralizing antibodies against FVIII develop in nearly 30% of the cases, leaving these patients with very limited therapeutic alternatives. Interestingly, Hemophilia B (OMIM # 306900) is clinically analogous to Hemophilia A, but correlates to deficient or dysfunctional FIX (Jaloma-Cruz et al., 2012). It has a prevalence of 1:25,000 males, and it is caused by mutations in the *F9* gene, at Xq27.1-27.2. The molecular mechanisms are heterogeneous and include a large spectrum of missense mutations (∼59%), frameshifts (∼15%) and splice site mutations (∼9%). FIX is a single-chain, vitamin-K dependent 57 kDa glycoprotein, synthesized by hepatocytes as a pre-pro-protein of 461 amino acids (aa) and it is structurally similar to both FX and FVII (Kah Yuen et al., 2017). As for Hemophilia A, the treatment option mainly relies on replacement therapy, based on purified, recombinant concentrates or Prothrombin Complex Concentrate (PCC). In contrast, the incidence of the other bleeding disorders is around 1:1,000,000 and 1:2,000,000 in the general population. Therefore, these are referred to as Rare Bleeding Disorders (RBDs) and are typically orphan diseases. Coagulation FVII deficiency (OMIM #227500) is recognized as the most common among the RBDs, with an estimated prevalence of symptomatic individuals of about 1:500,000 (Mariani and Bernardi, 2009; Napolitano et al, 2017).

### 3.2 FVII deficiency

FVII deficiency is a rare monogenic, recessive disease resulting from an heterogenous pattern of mutations in the F7 gene, located at 13q34. It is estimated that around 78% of the disease-associated mutations are missense mutations associated with reduced FVII plasma activity in affected individuals. FVII is a vitamin K-dependent serine protease produced by the liver parenchyma as a 50 kDa multidomain glycoprotein. Native FVII consists of 406 aa, composing a N-terminal γ-carboxyglutamic acid domain followed by two epidermal growth factor-like domains (EGF1 and EGF2), and a C-terminal protease domain (Monroe and Key, 2007). It is a unique factor that includes a fraction of around 3% circulating in an active form, even in the absence of coagulation activation. As previously mentioned, FVII plays a fundamental role in coagulation, initiating coagulation upon interaction with TF. FVII deficiency is characterized by a wide, heterogeneous pattern of clinical phenotypes, ranging from asymptomatic to severe and lethal hemorrhagic forms, even in the presence of the same levels of FVII coagulant activity. The most frequent clinical manifestations are mild, whereas 15% of the affected individuals exhibit severe, life-threatening bleeding episodes. The recurrent bleeding symptoms are similar to those presented in platelet disorders, with frequent epistaxis, menorrhagia, post-operative bleeding, and bruising. The severe disease phenotype is clinically defined as FVII activity below 10%, correlating with higher risk of recurrent and intense bleeding, while the moderate form, 10%–20% and the mild deficiency from 20% to 50% activity. In severe cases, Central Nervous System (CNS) and Gastrointestinal (GI) hemorrhages are relatively common, as well as joint bleeding and hemarthrosis-related arthropathy (Mariani et al., 2005). As for the other bleeding disorders, the current treatment is based on replacement therapy with highly expensive and frequent injections of exogenous FVII concentrates, either purified from plasma (pdFVII) or produced by recombinant technology (rFVII). Unfortunately, rFVII requires frequent bolus injections and careful monitoring due to its short half-life (<3 h).

## 4 Liver architecture

**FIGURE 2:**
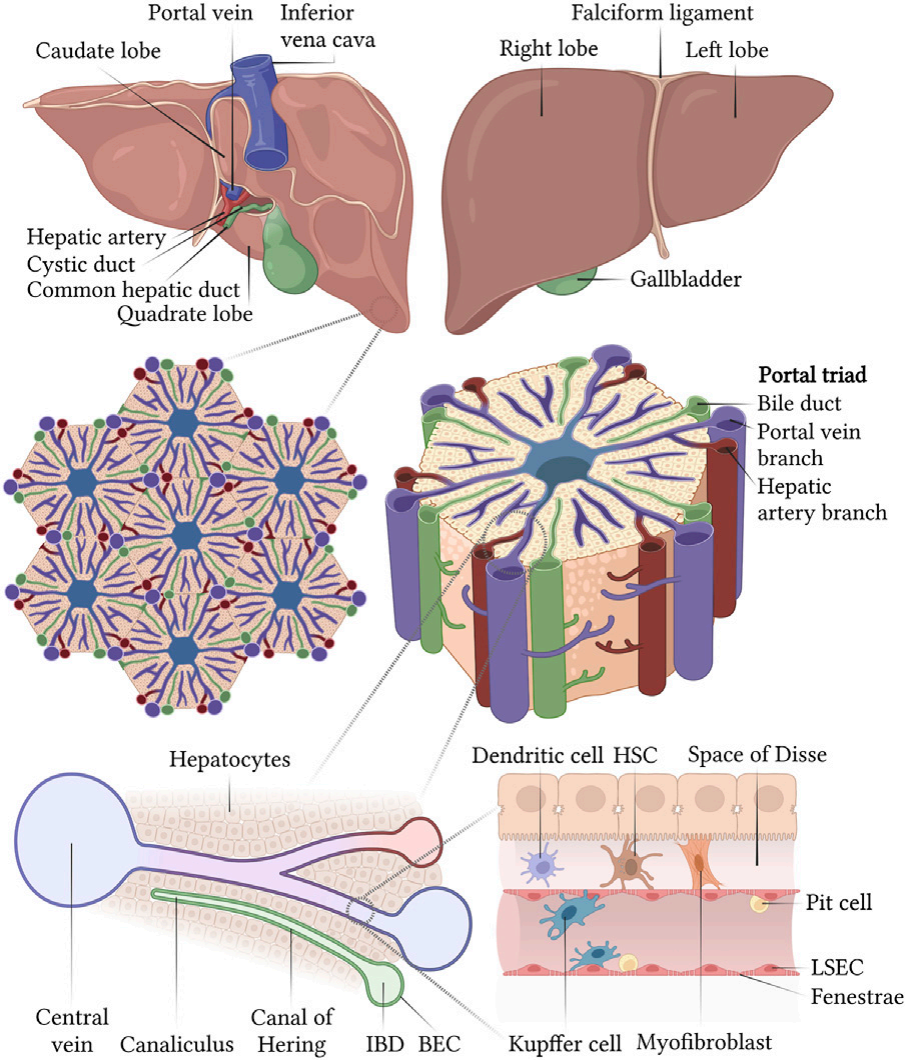
Structural organization of the liver at different scales and schematic representation of a liver sinusoid. Created with BioRender.com.

A direct evidence of the organ- and hepatocellular specificity of the blood clotting factors has been obtained in the past years from hepatic injury and dysfunction evaluations (Mannucci and Tripodi, 2012), primary hepatocytes in culture (Boost et al., 2007; Tatsumi et al., 2008; Biron-Andréani et al., 2010) and liver perfusion studies (Olson et al., 1966). These studies show that most of the anticoagulant and procoagulant factors, except FIII, FVIII and vWF, are synthesized and secreted by hepatocytes, within the liver parenchyma. The primary site of FVIII synthesis, however, has been attributed to the Liver Sinusoidal Endothelial Cells (LSECs) (Fahs et al., 2014). The liver is the largest endocrine organ in the human body responsible for the maintenance of blood homeostasis and hemostasis. In addition to bile production, regulation of glycolytic, urea and cholesterol metabolism, it serves in xenobiotic metabolism, hormone synthesis and plasma protein secretion. The smallest functional unit within the liver is the portal lobule, comprising a polygonal columnar segment of highly polarized hepatocytes, connected to form the hepatic lamina and arranged in a radial pattern around the central vein (Slim et al., 2014) (Figure 2). Hepatocytes represent ∼60% of the total population and ∼80% of the total hepatic mass (Liu and Crawford, 2004). The hepatic lamina and the bloodstream are in contact via the space of Disse, lined by the LSECs, accounting for ∼50% of the total liver Non-Parenchymal Cells (NPCs), in the form of a porous capillary network connecting with the central vein (Fujiyoshi et al., 2021). In addition to the LSEC, the sinusoidal NPCs comprise of 35% of the total hepatic population and include the Hepatic Stellate Cells (HSCs), Kupffer cells and hepatic Natural Killer (NK) cells (Vekemans and Braet, 2005). HSCs, or Ito cells, are quiescent pericytes with long dendritic processes found in the space of Disse which are involved in the maintenance of the microenvironmental homeostasis, and are activated during fibrosis and oxidative stress events by the resident tissue macrophages, the Kupffer cells (Feldman et al., 2010). In contact with the LSEC and, frequently, with the Kupffer cells, the hepatic NK cells, or Pit cells, are resident, liver-specific NK cells with a large and granular morphology. In addition to the Kupffer and hepatic NK cells, the immune, liver-specific NPCs include two other cell types, the hepatic dendritic cells and NK T (NKT) cells, innate-like lymphocytes recognizing lipid antigens and are enriched in the microvascular compartments. The basolateral surface of hepatocytes interacts to form a highly branched luminal network, a continuous canaliculus that is accounted for bile secretion and draining toward the Intrahepatic Bile Ducts (IHBD). These IHBDs are composed of cholangiocytes, a heterogeneous and dynamic cell population lining the intrahepatic biliary tree that modulates the bile composition (Tabibian and LaRusso, 2014).

## 5 iPSC-derived liver organoids: From modeling to the treatment of bleeding disorders

Several limitations of the current hepatic models have hindered the study and treatment of inherited coagulation factor deficiencies. When cultured *in vitro*, primary human hepatocytes (PHHs) exhibit a dramatic impairment of the proliferation capacity and a rapid loss of both hepatocellular morphology and functionality, as is also the case for primary HSCs, KCs and LSECs (Dusabineza et al., 2015; Olgasi et al., 2020). The progressive de-differentiation of PHHs is accompanied by the failure in re-establishing the unique hepatic cell polarity and metabolic activity, caused by the disruption of the Cell Adhesion Molecules (CAMs) during the isolation procedures and conventional culturing in 2D monolayer (Zeigerer et al., 2017). In this regard, different protocols have succeeded in generating hepatocyte-like cells from induced pluripotent stem cells (iPSCs) in the form of 2D culture systems and 3D organoids, with the purpose of studying liver functions and liver disease (Rambhatla et al., 2003; Schwartz et al., 2006; Cai et al., 2007; Hay et al., 2007; Söderdahl et al., 2007; Subramanian et al., 2014). iPSCs are somatic cells reprogrammed into an embryonic-like pluripotent state, through the ectopic expression of Oct4, Sox2, Klf4 and c-Myc (the Yamanaka factors), a set of transcription factors responsible for self-renewal and pluripotency (Takahashi and Yamanaka, 2016). It is possible to reprogram adult, patient-derived cells, such as fibroblasts, CD34^+^ Hematopoietic Stem and Progenitor Cells (HSPCs) and Peripheral Blood Mononuclear Cells (PMBCs), using feeder-free, and non-integrative, methods such as episomal or Sendai virus (Malik and Rao, 2013). The embryonic-like pluripotent state enables iPSCs to self-renew indefinitely and to differentiate into all three germ layers, endo-, meso- and ectoderm, thereby providing an unlimited source of virtually any human cell type required for modeling or therapeutic purposes. Several methods have been developed during the last decades to manipulate and mimic the factors orchestrating embryonic organogenesis to replicate *in vivo* developmental signals and guide the stepwise differentiation of iPSCs toward 2D and advanced 3D cell structures. Based on the accumulated knowledge of liver embryonic development, several recent studies have developed increasingly efficient protocols that resemble the different stages of *in vivo* hepatogenesis in a specific spatial-temporal manner (Guan et al., 2017; Koui et al., 2017; Zhu et al., 2017; Ouchi et al., 2019; Wu et al., 2019). As a result, 3D cultures have been established to mimic the structural architecture and features of the hepatic microenvironment, by utilizing the spontaneous propensity of iPSCs to self-aggregate in a suspension culture upon single cell seeding. These 3D aggregates, termed HOs, are *in vitro* culture systems of multiple, self-assembled and self-organized hepatic cell types, recapitulating a complex structural and functional microscale layout *in vivo* (Rozich et al., 2019). Recently, pioneering studies in the Sullivan laboratory have established a scalable, small molecule-based, extracellular matrix (ECM)-independent approach to generate functional HOs. These have enhanced function and maturity, sinusoid-like vascular structures, a complex liver-like cellular repertoire and *de-novo* innervation (Harrison et al., 2020). Thus, novel strategies have recently been established for the production and secretion of the variety of coagulation factors, and their use in personalized, iPSC-based technologies. In this sense, pioneering studies have only recently disclosed the unprecedented potentials of HOs for therapeutic, translational purposes in this unmet medical need of coagulation factor deficiencies. As an example, a study conducted by Pettinato et al. (2019) has described highly functional hepatocyte-like clusters differentiated from iPSCs co-cultured with Human Adipose Microvascular Endothelial Cells (HAMEC), in a 3D culture system. Here, they observed sustained production and secretion of all the coagulation factors, over time. Surprisingly, Pettinato et al. (2019) demonstrated how this novel activity was out performing PHHs after only 4 days, due to de-differentiation *in vitro*. In particular, antigens of disease-relevant factors such as FVII, FVIII, vWF, were at physiological levels, when compared to the normal secretome profiles of PHHs (Pettinato et al., 2019). As such, these findings have opened the doors to the establishment and optimization of novel cell-therapy applications of HOs in the field of inherited coagulation factor deficiencies. Among the most relevant and recent studies, Harrison et al. (2020) have generated iPSC-derived HOs containing a complex hepatocellular repertoire, which recapitulate the *in vivo* heterogeneity and organotypic architecture of human liver. The synthesis and secretion of all the coagulation factors and inhibitors, including the vitamin K-dependent FX, VII, prothrombin, Protein S and C, AT were reported to be comparable to PHHs, but importantly maintained over time. In agreement with Subramanian et al. (2014), this is driven by the fact that the self-assembly process and the tissue-like 3D structure of HOs exponentially enhances the hepatic specification, resulting in comprehensive and proficient *in vivo*, liver-like functional attributes. Indeed, these HOs include the whole spectrum of liver NPCs and present with extensive parenchymal cell polarity with the establishment of partial hepatic zonation, and broad *de novo* vascularization and innervation (Harrison et al., 2020; Olsen et al., 2021). Since it has a key role in coagulation, Harrison et al. (2020) have also closely assessed the antigen and activity levels of FVII present in the HO medium. They have observed physiological, therapeutic levels of FVII which correlated with efficient activity and inducibility of the *in vivo* hepato-metabolic competence in the long term. The ground-breaking, therapeutic relevance of these iPSC-based HOs does not only rely on the maintenance of the factor’s activity comparable to PHHs, but also the patient-specific nature of the approach.

### 5.1 Hemophilia B

As a consequence, two recent pioneering studies had the ambition to investigate HOs generated from Hemophilia patients where the disease-causing mutation was corrected using CRISPR/Cas9 technology. Their aim was not only to model the disease but also treat the disease-phenotype by long-term engraftment. Luce et al. (2021) reported the generation of HOs from a severe Hemophilia B (HB) patient, carrying the g.31280 G>A (c.1297G>A, p.E433K) mutation. In particular, this alteration leads to severe FIX inactivation, which is clinically characterized by FIXc <1% and FIXAg 67% levels respectively (Luce et al., 2021). Since conventional 2D differentiation displayed incomplete *in vitro* hepatic maturation, the patho-molecular mechanisms underlying the g.31280 G>A mutation have been investigated using a novel HO model. Consequently, HOs have proven a unique platform to investigate and uncover the molecular and intracellular mechanisms involved in disease pathogenesis, since they harbor the exact constellation of genetic variants present in the parental patient-derived iPSCs (Lancaster and Huch, 2019). Several studies have demonstrated the versatility of CRISPR/Cas9 in precisely editing exonic non-synonymous single nucleotide variations (nsSNVs) as well as large insertions, and this technology is particularly useful in the treatment of coagulation factor deficiencies (Lyu et al., 2018; Roper and Yilmaz, 2019; Son et al., 2022). Accordingly, syngeneic disease and non-disease models can be generated by either correcting coagulation deficiency-affected iPSCs or re-creating relevant point mutations, consequently overcoming the obstacle of sample accessibility and experimental variability (Hockemeyer and Jaenisch, 2016; McTague et al., 2021). In addition, the iPSC-based, CRISPR-edited system may allow the modification of a single allele, thereby recapitulating the genetic background of a heterozygote (Rabai et al., 2019; Prat et al., 2020). This strategy has been adopted by Luce et al. (2021) where they have generated syngeneic disease and disease-corrected models to investigate HB and to pave the way for the next experimental phases, consisting of gene editing and cell-therapy for HB. In additional studies performed by Lyu et al. (2018), it has been recently demonstrated that it is possible to stably restore FIX secretion upon integration of a therapeutic copy of F9, *via* the Adeno-Associated Virus Integration Site 1 (AAVS1) locus, using CRISPR-mediated Homology Directed Repair (HDR). Based on these findings, Luce et al. (2021) then moved far beyond the initial modeling purposes, by introducing a therapeutic F9 minigene into HB iPSCs carrying the Padua mutation (g.31134 G>T, p.R384L) to enhance FIX activity, prior to HO differentiation. When using standard 2D recombinant technology and culture system, Luce et al. (2021) reported dramatic and substantial differences in size, half-life and activity of the recombinant FIX protein. This was caused by complex, *in vitro* post-translational modifications, which were responsible for an abnormal lack of γ-carboxylation of the GLA-rich domain. However, in HOs where the mutation was corrected, the recombinant FIX protein was the same size, contained the expected post-translational modifications and global clotting activity of the plasmatic protein (Luce et al., 2021). For this reason, they transplanted the corrected HOs into newborn HB, F9 KO mice and monitored them for 2 weeks. They demonstrated that the F9 minigene knock-in could rescue FIX physiological levels and activity, thus reversing the disease phenotype in F9-KO mice. Interestingly, these preliminary findings overcame one of the relevant challenges presented in the previous studies of Ramaswamy et al. (2018), describing drastically limited therapeutic effects and compromised engraftment when using conventional 2D iPSC-derived hepatocyte-like Cells (HLCs). Luce et al. (2021) also highlight that single-cell techniques led to dramatically low performances in terms of hepatic maturation, proliferation, transplantation, and therapeutic relevance. As a consequence, they addressed the need of novel 3D approaches, to circumvent these limitations and give a long-term, therapeutic solution to HB. In this regard, the study is strongly limited by a short-termed experimental layout, and definitely requires long-term analysis in alternative animal models, such as immunocompromised, F9-KO mice.

### 5.2 Hemophilia A

Another pioneering study has been carried out by Son et al. (2022), achieving long-term therapeutic correction of Hemophilia A. As a proof-of-principle they have demonstrated that CRISPR/Cas9 technology could correct Inv22 in patient-derived iPSCs. Here, HOs from genome-edited iPSCs were transplanted into hemophilic mice, where they detected secretion of significant amounts of functional FVIII, followed by a dramatic reversion of the bleeding phenotype. Based on these findings, they hypothesized that the liver-like, multi-cellular repertoire of HOs could provide an optimal microenvironment for resident endothelial cells to boost and sustain corrected-FVIII expression over time (Son et al., 2022). In addition, they anticipated how multicellular 3D liver organoids could enable a more effective correction of HA when compared to traditional 2D techniques, by reducing the number of cells to engraft and enhancing the expression of functional FVIII (Son et al., 2022). They calculated that engraftment of ∼2800 HOs, containing 4*10^^6^ endothelial cells was sufficient to achieve a therapeutic outcome (Son et al., 2022). However, the study is limited to only 2 weeks evaluation, and by the use of immunocompetent HA mice treated with cyclosporine A.

### 5.3 The future of liver organoid-based therapies

**FIGURE 3:**
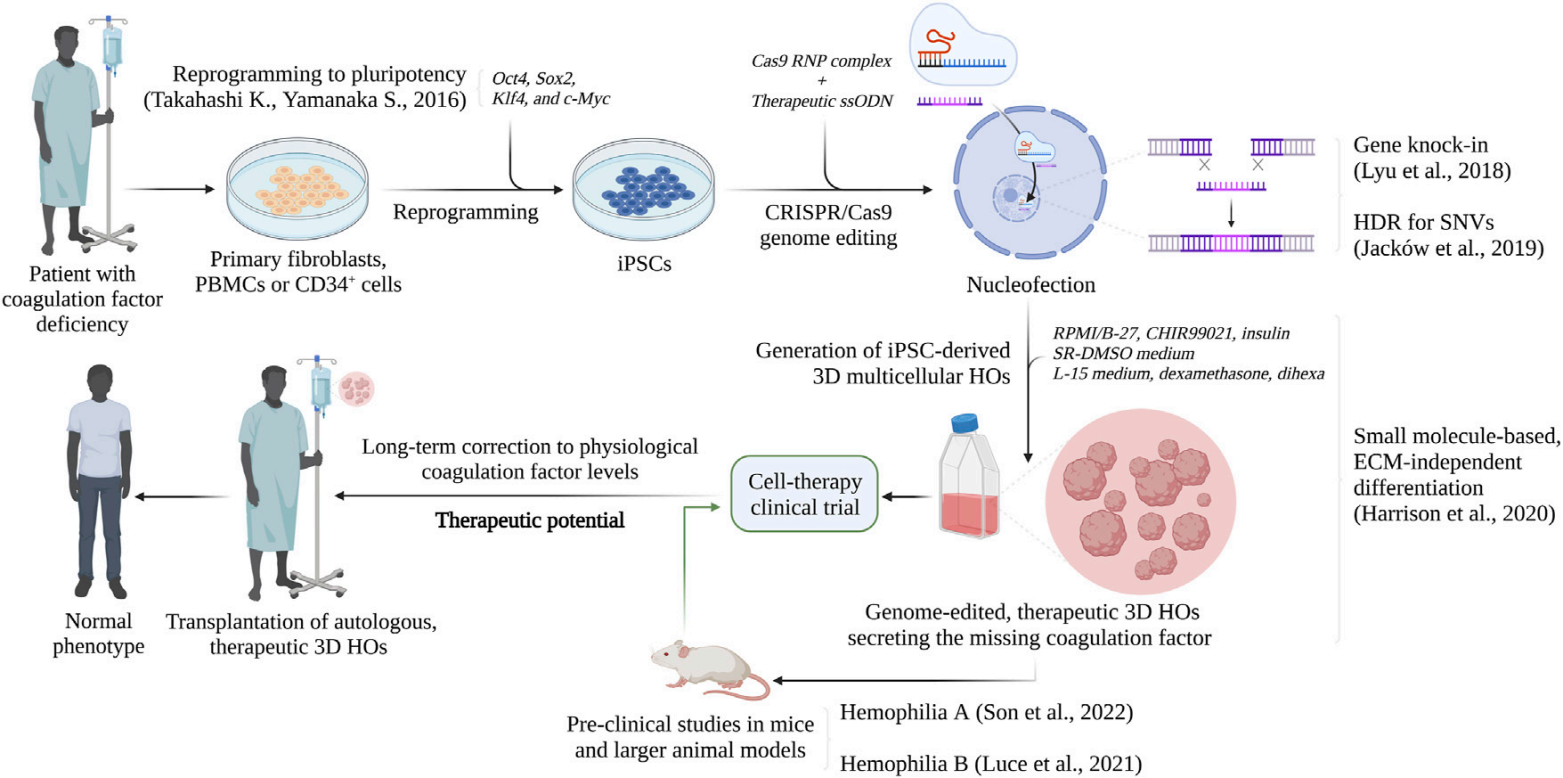
Future perspectives: the therapeutic potential for the development of cell-based therapies for inherited bleeding disorder. Created with BioRender.com.

As presented, recent studies have addressed the possibility to deliver genome-edited, patient-derived HOs through feasible, autologous transplantation and investigate these cells for their ability to revert the disease phenotype in a spectrum of bleeding disorders and liver diseases (Hockemeyer and Jaenisch, 2016; Son et al., 2022). Especially recent studies by Lyu et al. (2018), Ramaswamy et al. (2018), Luce et al. (2021) and Son et al. (2022) have laid the foundation for novel cell therapy to be potentially realized clinically for the treatment of coagulation bleeding disorders, in particular Hemophilia, in addition to liver disease (Figure 3). They have demonstrated how genome-edited, highly functional, iPSC-derived HOs can completely revert the bleeding phenotype of Hemophilic mice. However, these preliminary, pre-clinical achievements are limited to short-timed investigations, due to the lack of immunocompromised, KO mice. It is therefore an urgent need of studies exploring the therapeutic potential of HOs, and the survival and tolerogenic competency of the preclinical models in a long-term perspective. Several studies and clinical trials have described an unexpected role of hepatocytes and LSECs in modulating the immune response, by suppressing CD8^+^ and CD4^+^ T cell activation, and regulatory T cells (Tregs) (Arruda and Samelson-Jones, 2016; Crispe, 2016; Pratt et al., 2020). This may be one of the reasons behind the unprecedented therapeutic relevance of HOs, which is corroborated by the *in vivo*-like nature and hepatocellular heterogeneity of these cells. However, concerns regarding the safety of genome editing, particularly upon delivery *in vivo* or transplantation must be taken seriously. Thus, this is currently under major consideration, and several studies have recently demonstrated the effectiveness and, particularly, safety of genome editing in a broad spectrum of hematological and coagulation diseases (Hoban et al., 2016; Ou et al., 2016; Ohmori et al., 2017; Lyu et al., 2018; Ramaswamy et al., 2018; Cosenza et al., 2021; Son et al., 2022). Importantly, clinical trials implementing CRISPR-edited HSPCs for the treatment of severe Sickle Cell Disease (SCD) and β-thalassemia are currently on-going, with no reported off-target effects (Frangoul et al., 2021). In the case of β-thalassemia, the U.S. Food and Drug Administration approved Zynteglo^®^, a one-time, single-dose gene therapy product to treat adult and pediatric β-thalassemia patients who require regular transfusions, in August 2022. With a prodigious 89% success rate, consisting of transfusion independence for at least 12 months, no signs of malignancies have been reported (FDA, 2022). The major concern of these treatments is that unintended, non-specific modifications may arise at locations other than the targeted site, due to sequence similarity (Zhang et al., 2015). However, several generations of Cas9 nucleases and accumulating protocols have maximized the HDR competency, while drastically abolishing residual side effects. In addition, extra precautionary measures are normally done where genome-edited iPSCs are characterized *ex-vivo* and examined for absence of off-target edits, homozygous or heterozygous correction occurrences, genome integrity and pluripotency, prior to differentiation toward HOs and transplantation (Jacków et al., 2019; Geng et al., 2020). This allows for the generation of safe, stepwise protocols to ensure and validate the long-term safety of the patients. When compared to the several limitations with standard 2D approaches, HOs have shown an exceptional ability to engraft into the kidney capsule of mice, either with or without ECM, and be maintained over time (Roper et al., 2017; Harrison et al., 2020; Son et al., 2022). The kidney is commonly considered the preferential administration route for HOs, since it is easily accessible and highly vascularized, and the retroperitoneal location allows advanced imaging and biopsy (Shultz et al., 2014). This method relies on the groundbreaking studies of Takebe et al. (2013), who was the first to establish iPSC-derived vascularized embryonic Liver Bud (iPSC-LB) organoids composed of Umbilical Cord Vein Cells (HUVECs), Mesenchymal Stem Cells (MSCs) and iPSC-derived hepatocytes. Following transplantation in immunocompromised mice, the vasculature of iPSC-LBs connected to the host vessels, thus allowing an efficient engraftment and the development of proliferating hepatoblasts into mature, highly functional hepatic tissue (Takebe et al., 2013). Consequently, this has demonstrated that it is possible to rescue drug-induced liver failure without the recipient liver replacement (Espejel et al., 2010; Takebe et al., 2013). However, obtaining a clinically relevant HO engraftment in humans is challenged by several considerations, particularly in terms of cell number and survival. Indeed, the minimal scale required for a potential clinical translation is generally identified as 10^^9^ hepatocytes per single adult patient (Horslen and Fox, 2004; Olsen et al., 2021). Most of the current approaches are still set up for the pre-clinical scale, and not yet optimized for the industrial scale and human application. In this perspective, the recent advances in increasingly optimized and standardized differentiation protocols, as presented by Harrison et al. (2020), and in the use of bioreactors may exponentially boost the establishment of an effective and safe bioprocessing, as well as future clinical trials. As presented by Luce et al. (2021), a possible alternative to refine the amount of cell needed for therapeutic purposes may be the use of strategies to enhance protein expression. It is critical to highlight that coagulation factor deficiencies can be corrected or improved from severe to mild clinical presentations even by minor increases in the protein levels and activity. However, there are many obstacles to overcome in order to clinically translate the potential of these novel iPSC- and HO-based therapies. Olsen et al. (2021) have observed that the *in vitro* differentiation of iPSCs to organoids correlate with low degrees of inflammation, which often results into batch variability and HSC-like cell stimulation. For this reason, a further optimization of the current culture conditions is needed. Furthermore, genomic stability, fluctuation in the reprogramming quality and functional pluripotency among iPSC lines, and residual iPSCs in the HOs to be transplanted should be taken into consideration (Son et al., 2022). To address this, increasing stepwise validation methods have been developed in order to examine the quality, the content, and the safety of HOs. While the genome integrity of iPSCs and, particularly, CRISPR-edited iPSCs is validated by whole genome DNA sequencing, HOs are carefully interrogated *via* proteomic analysis and single cell sequencing for the absence of any residual iPSC, to completely eradicate the risk of teratomas. Also, such analyses are needed to confirm the identity and quality of the cell subtypes present in the HOs, to ensure the safest and most efficient therapeutic benefit. Another significant limitation in cell therapy is the graft rejection, caused by the immune host reaction as a consequence of a non-compatible or partially mismatched Human Leukocyte Antigen (HLA), or Major Histocompatibility Complex (MHC) (Madrid et al., 2021). Consequently, patients’ iPSC-derived HOs are autologous and circumvent this obstacle, thus allowing for long-term engraftment without an adverse reaction or the need of immunosuppressive regimens. To investigate this further, several clinical trials for autologous iPSC-based transplantations have recently validated the absence of side-effects after 5 years of follow-up (Palta et al., 2014; Takagi et al., 2019; Schweitzer et al., 2020; Madrid et al., 2021). Numerous studies in the last decade have reported with success the complete absence of immune rejection after autologous iPSC-derived cell therapy in animal models (Araki et al., 2013; Guha et al., 2013; Osborn et al., 2020; Schweitzer et al., 2020). To overcome these challenges, further investigations are necessary and the use of larger animal models, such as pigs, are required in order to define the right dose per patient, the survival of engrafted HOs, and the long-term therapeutic outcome, and the tolerance of the transplant recipient, prior to clinical trial assessments expected in the coming years.

## 6 Conclusion

Several efforts have been made during the last decades in order to disclose and recapitulate the signaling pathways governing the *in vivo* fetal development, and in particular hepatogenesis. These findings have been gradually translated into innovative, defined protocols that have been optimized in order to generate an unrestricted number of different hepatic cells, needed for both research and therapeutic applications. By providing the key growth factors or small molecule mimetics in a dose- and time-specific manner pioneering studies have succeeded in patterning iPSCs into functional hepatocytes, in both 2D and 3D cultures. These models have served to primarily study liver physiology as well as developing models for liver disease. However, several studies have recently started to use these models to investigate the production of coagulation factors proteins, with very promising results (Pettinato et al., 2019; Harrison et al., 2020). In contrast to the conventional 2D approaches, which may lead to recombinant proteins devoid of proper post-translational modifications, the HOs have succeed in secreting identical plasmatic proteins, correlating with a proficient, physiological clotting activity (Luce et al., 2021). Since severe bleeding disorders are life-threatening and they are induced by the deficiency or reduction of activity of blood coagulation factors, there is a need for a correction of factor function. In particular, these disorders correlate with spontaneous and acute bleeding episodes, such as intracranial and joint bleedings, which in turn lead to a dramatically decreased quality and life expectancy. Moreover, the only option for treatment consists of protein replacement, which often results in the development of inhibitors and a more complicated handling of these patients. As a consequence, novel therapeutic strategies are needed. To fill this void, different studies have recently investigated and disclosed the unprecedented potentials of HOs which secrete all the coagulation factors at physiological level long term (Pettinato et al., 2019; Harrison et al., 2020). Generating HOs from patient-derived iPSCs is a state-of-the-art, powerful tool for disease modeling, but it is only one of the limitless possibilities of this novel technology. However, considering that proof-of-principle studies have been presented only for HA and HB, application of HOs still needs to be verified and extended to the other coagulation factor deficiencies, such as FVII deficiencies and the other rare, inherited coagulation disorders with large unmet medical need. As such, this approach will also provide a therapeutic alternative to pediatric patients who are not eligible for the standard gene therapy, such as the current AAV-based techniques correlating with possible therapeutic failures due to liver growth (Luce et al., 2021).

In conclusion, the combination of induced pluripotent stem cell (iPSC) technology and HOs with the CRISPR/Cas9-based genome editing has opened the doors for pioneering paradigms for regenerative medicine within blood coagulation factor deficiencies and liver diseases. The outcome is a groundbreaking model for use in countless basic, translational, and pre-clinical research applications, particularly relevant in the field of coagulation disorders.
